# From rare to well-done: importance of rare tumors in cancer therapeutic advances

**DOI:** 10.18632/oncotarget.27020

**Published:** 2019-06-18

**Authors:** Florence Duffaud, Sophie Piperno-Neumann, Nicolas Penel, Jean-Yves Blay

**Affiliations:** ^1^ Oncology Medical Unit, CHU La Timone and Aix-Marseille University, Marseille, France

**Keywords:** osteosarcoma, regorafenib, TKI (tyrosine kinase inhibitors)

Osteosarcomas represent one of the earliest cancers to prove the beneficial impact of empiric cytotoxic chemotherapy, but there has been little to no progress in improving outcomes for this uncommon subtype of sarcoma over the past three decades. Treatment options for metastatic and/or recurrent osteosarcoma are woefully inadequate, and these patients, whether pediatric or adult, have an overall poor prognosis. Our field understands very little about why some patients with osteosarcoma can be cured with chemotherapy, while others not. New insights to the biology of malignant bone tumors are needed, and clinically validated mechanisms to build upon are necessary to advance beyond the gains of empiric chemotherapy. This is why the activity of a multi-targeted (i.e. semi-selective) kinase inhibitor drug in patients with recurrent/metastatic osteosarcoma following failure of standard chemotherapy is so important to the field and to cancer research in general.

The French Sarcoma Group (FSG) recently published an investigator-initiated, randomised, double-blind, placebo-controlled, multicentre phase II study [1] that evaluated efficacy and safety of regorafenib in adult patients with metastatic osteosarcoma after failure of previous chemotherapy. After 8 weeks, the disease control rate (non-progression rate) was 65% in the regorafenib group compared to 0% in patients initially randomly assigned to placebo; although technically a non-comparative, signal-seeking study, this qualified as having successfully met the trial’s primary endpoint. The metastatic osteosarcoma remained under control after 6 months in 35% of the patients receiving regorafenib. Other analyses, including a longer time to progression after cross-over to regorafenib after initial progression on placebo, also support the clinical activity of this agent in these patients.

Although osteosarcoma remains a rare disease, this study shows that it is feasible to perform signal-seeking studies to detect activity based on rates of disease control with concurrently randomized patients. The possibility of cross-over at progression for patients randomized to placebo (or some standard treatment) to receive the new drug makes the randomized controlled phase 2 trial design psychologically more acceptable to physicians, patients, parents, and ethical review committees. Of course, any sort of cross-over study design confounds the ability to assess any impact of a new treatment on overall survival; however, signal-seeking studies should seek more direct near-term data on drug effect, so overall survival is appropriate to leave for later trials.

The heterogeneity of osteosarcoma population supports the need for randomised Phase II, especially when using PFS as primary end-point, as recommended by experts [2, 3], as a possible more efficient end-point in recurrent osteosarcoma Phase II studies, considering as events disease progression at any site and death from any cause.

This FSG study was amended to enable inclusion of adolescents and children patients (age ≥ 10 years old). As recommended by the ACCELERATE Fostering Age Inclusive Research (FAIR) trial group [4] supported by FDA [5], for rare adult disease present in adolescents, the adolescent population should be included in adult initiated trial to accelerate new drug access. Interestingly, not a single child with osteosarcoma was enrolled in the study, likely because of the inclusion of the placebo arm.

It is of interest that many pediatric osteosarcoma experts now feel that an uncontrolled study design might be sufficient to assess a promising signal of anticancer activity from new agents in this disease [6]. Consistent with this, it is not that the results of this randomized trial are rather consistent with results from other uncontrolled phase 2 studies in relapsed and unresectable osteosarcoma with other multi-kinase inhibitors; these other trials have reported median of progression-free survival (PFS) of 4 months (95% CI 2–5) [7], 5 months (95% CI 2–7) [8], 6.2 months [9], and 4.5 (95% CI 3.47–5.27) months [10], with, sorafenib alone, sorafenib and everolimus, cabozantinib and apatinib, respectively, (with PFS rates at 6 months of 29%, 33%, 36.7%, and 45% with sorafenib, cabozantinib, apatinib, and sorafenib+everolimus respectively). Thus, it is possible in a severe, life-threatening metastatic disease such as progressive osteosarcoma after failure of prior chemotherapy to have reasonable confidence in the assessments of uncontrolled trials, judging from the totality of this evidence.

The mechanism(s) by which these kinase inhibitors with pleiotropic activity on a number of key kinase pathways may slow progression of chemotherapy-resistant osteosarcoma remains obscure and is a reasonable subject for further scientific and medical inquiry. Building on these results, it may be of interest to move such kinase inhibitor therapy earlier in the course of disease, for example in an effort to improve cure rates in the adjuvant disease treatment setting of very high risk individuals. Such trials are feasible since the osteosarcoma community has a long history of conducting cooperative trials despite the rarity of this disease, building on an established clinical research network which links groups around the world.

In summary, regorafenib demonstrates meaningful activity in adult patients with recurrent, progressive, metastatic osteosarcomas after failure of conventional chemotherapy, with positive impact in delaying disease progression. Although the specific molecular mechanism(s) remain unclear, regorafenib might have an important therapeutic role as an agent complementary to standard cytotoxic chemotherapy or as an adjunct to modern immune-oncology approaches, which have, to date, been negative in osteosarcomas. Building off positive data offers hope for the future of medical science, but also real hope for patients in need of advances to improve outcomes.
